# Supplementary material for: DNA barcoding and species delimitation of the Old World tooth-carps, family Aphaniidae Hoedeman, 1949 (Teleostei: Cyprinodontiformes)
Source: PLoS One. 2020 Apr 16;15(4):e0231717. doi: 10.1371/journal.pone.0231717 (PMC7162479; doi:10.1371/journal.pone.0231717)
Supplement: S1 Appendix — (DOCX) [file pone.0231717.s001.docx]

**Material examined:**

***Aphanius arakensis:*** ZM-CBSU 10999, holotype, 1, 31 mm SL; –ZM-CBSU 11051, paratypes, 69, 22–34 mm SL; Iran, Markazi prov.: Namak Lake Basin at Arak, 34°00'N; 49°50'E.

***Aphanius darabensis*:** ZM-CBSU 9713, holotype, 1, 24 mm SL; –ZM-CBSU 9601, paratypes, 68, 19–35 mm SL; Iran, Fars prov.: Kol River drainage, Korsiah Banaki spring, 28°46'24.96''N, 54°23'35.48''E. –ZM-CBSU 8683, 19, 15–28 mm SL; –ZM-CBSU 10871, 11, 13– 23 mm SL; Iran, Fars prov.: Kol River drainage, Dasht-e-Konar wetland, 28°09'23.1''N, 55°18'17.5"E.

***Aphanius farsicus*:** ZM-CBSU 9410, 66, 20–35 mm SL; Iran, Fars prov.: Maharlu Lake Basin, Barm-e-Shur spring, 29°27'N, 52°42'E.

***Aphanius isfahanensis*:** ZM-CBSU 6471, 31, 18–34 mm SL; Iran, Esfahan prov.: Zayanderh River near Varzaneh, 32°25'N, 52°39'E.

***Aphanius kavirensis:*** ZM-CBSU 9587a, holotype, 1, 27 mm SL; –ZM-CBSU 1141, paratypes, 59, 15–42 mm SL; Iran, Semnan prov.: Kavir Basin, Cheshmeh Ali Spring, 36°16'45.6'' N, 54°05'01.6'' E.

***Aphanius shirini:*** ZM-CBSU ZG151, holotype, 1, 34 mm SL; –ZM-CBSU ZG267, paratypes, 7, 21–35 mm SL; Iran, Fars prov.: Kor River Basin, Khosroshirin spring, 30°53′29.5˝ N 52°00′36.8˝ E.

***Aphanius sophiae*:** ZM-CBSU 8460, 70, 19–37 mm SL; Iran, Fars prov.: Kor River Basin, Ghadamgah spring, 30°15'N, 52°25'E.

***Aphanius vladykovi*:** ZM-CBSU 6401, 70, 16–41 mm SL; Iran, Chahar Mahale Bakhtyari Prov.: Karoun River drainage, Chaghakhor wetland, 31°55'N, 50°56'E.

***Aphaniops dispar:*** ZM-SBUK 101, 14, 26.2–37.9 mm SL; Djibouti,: Lake Assal, 11°40'36.6″N 42°27'17.2″E.

***Aphaniops furcatus:*** ZM-CBSU 225, holotype, 1, 21 mm SL; ZM-CBSU 211, paratypes, 14, 18–25 mm SL; Iran, Hormuzgan prov.: Shur River about 20km East of Bandar Abbas, 27°19''37.6'N, 56°28''10.2'E.

***Aphaniops ginaonis:*** ZM-SBUK 221, 10, 23.4–34.2 mm SL; Iran, Hormuzgan Prov.: Genow hot spring, 27°26'77.2″N E56°17'97.0″E.

***Aphaniops hormuzensis:*** ZM-FISBUK 157, holotype 1, 35.8 mm SL; –ZM-FISBUK 164, paratypes, 11, 22–34 mm SL; Iran, Hormuzgan Prov.: Mehran River at Gotab village, 27°80'39.8''N 54°150'46.1''E.

***Aphaniops richardsoni*:** ZM-FISBUK 120, 10, 22.2–34.6 mm SL; Jordan, Ain Abat, , 31°38' 63.4″N E35°30'32.3″E.

***Aphaniops sirhani*:** ZM-FISBUK 321, 4, 27.4–35.8 mm SL; Jordan, Azraq Wetland Reserve, 31°49'33.57″N 36°49'37.14″E.

***Aphaniops stoliczkanus*:** ZM-FISBUK 130, 10, 26.4–38.9 mm SL; Iran, Fars prov.: Howba spring, 27°57'30.5″N E53°53'58.4″E. –ZM-FISBUK 170, 10, Iran, Bushehr Prov.: Dalaki hot spring, 29°24'07.9″N 51°16'35.4″E. –ZM-FISBUK 173, 10, 27.5–40.6 mm SL; Khuzestan Prov.: Sartang spring, 29°24'07.9″N 51°16'35.4″E. –ZM-FISBUK 200, 16, 27.3–39.5 mm SL; Oman, Muscat prov.: coastal lagoon, Seeb city, 23°40'19.6″N 58°12'21.3″E. –ZM-FISBUK 220, 30, 26.6–38.3 mm SL; Iraq, Anbar prov.: Fallujah, 33°45'28.8″N 33°21'14.4″E.

***Paraphanius mento*:** ZM-FISBUK 401-402, 2, 24.2–26.8 mm SL; Lebanon, Beirut.
