# Supplementary material for: DNA barcoding and species delimitation of the Old World tooth-carps, family Aphaniidae Hoedeman, 1949 (Teleostei: Cyprinodontiformes)
Source: PLoS One. 2020 Apr 16;15(4):e0231717. doi: 10.1371/journal.pone.0231717 (PMC7162479; doi:10.1371/journal.pone.0231717)
Supplement: Attachment — Submitted filename: Response to Reviewers.docx [file pone.0231717.s003.docx]

**Dear Prof. Roberta Cimmaruta**

Academic Editor

Plos One

Many thanks for sending our manuscript PONE-D-19-33131R1
“DNA barcoding and species delimitation of the Old World tooth-carps, family Aphaniidae Hoedeman, 1949 (Teleostei: Cyprinodontiformes)” for the second round of revision. We also would like to thank the respected reviewer for his/her constructive comments on our manuscript. We found the comments and suggestions very constructive. So, we implemented all of them.

We also considerd the following issues for submitting our revised manuscript:

- A rebuttal letter that responds to each point raised by the academic editor and reviewer(s). This letter was uploaded as separate file and labeled 'Response to Reviewers'.
- A marked-up copy of the second revised manuscript that highlights changes made to the original version. This file was uploaded as separate file and labeled 'Revised Manuscript with Track Changes'.
- An unmarked version of of the second revised paper without tracked changes. This file was uploaded as separate file and labeled 'Manuscript'.
- The figure files were uploaded to the Preflight Analysis and Conversion Engine (PACE) digital diagnostic tool, <https://pacev2.apexcovantage.com/> to be ensured that figures met PLOS requirements.

We also made very few minor grammatical corrections. These are clearely indicated using Track Change System.

I hope this revised manuscript is now in order and shall find a place in the forthcoming issue of the Plos One journal.

One again, I ihank you for handling the ms.

Sincerely Yours

**Details of the answer to the comments:**

PONE-D-19-33131R1
“DNA barcoding and species delimitation of the Old World tooth-carps, family Aphaniidae Hoedeman, 1949 (Teleostei: Cyprinodontiformes) ”

**The main points suggested by respected reviewer:**

#Line 203. Change “Sampling and morphological identification” to “Sampling and external morphological identification”

ANSWER: Corrected: It was changed to: Sampling and external morphological identification

#If meristic counts of fin rays were not done in cleared and stained specimens, please change: “based on the external morphology, including coloration and meristic counts” to “based on the external morphology and including coloration.” (lines 207-208).

ANSWER: Corrected: It was changed to “based on the external morphology and including coloration”

#Then add to the end of that paragraph “Meristic counts were done externally (not using cleared and stained specimens).” (line 210).

ANSWER: Corrected: the following sentence was added to the end of paragraph.

“Meristic counts were done externally (not using cleared and stained specimens)”

To the knowledge of this reviewer, fin rays count should always be done in cleared and stained specimens and not externally.

#Line 275. Add “belonging to morphologically identified species based on external characters”

ANSWER: It was in line 220:

Corrected: belonging to morphologically identified species based on external characters”

#As I mentioned in my previous review, every manuscript should be self-contained. Please include temperature cycle and which Taq/reagents/mastermix was used for PCR (lines 223-225).

ANSWER: The following sentence was added:

The amplification process was performed using Master Mix in a total volume of 25 μl containing 12.5 μl of a Ready 2X PCR Master Mix (Genetbio, Cat. no. G-2000), 0.5 μl of each primer (10 pmol/μl), 5 μl of the DNA template and 6.5 μl dd water. The amplification was performed on a Bioer XP Thermal Cycler (Bioer Technology Co. Ltd., Hangzhou, China), programmed as following: an initial denaturation at 94 °C for 3 min, 35 cycles with denaturation at 94 °C for 50 s, annealing at 52 °C for 1min, and a final extension phase at 72 °C for 5 min.

#Lines 239-241. Please move the phrase “The nucleotide substitution pattern showed that the sequences have not reached substitution saturation and are therefore well applicable for phylogenetic analyses.” to results section.

ANSWER: Done: the phrase was moved to the results section under the heading of **Molecular species delimitation (**lines 299-300).

#In my previous review I mentioned that considering the fossil calibration used (maximum age 34 Ma) (lines 307-309), the results should be cautionary interpreted. As no mutation rate has been reported for COI in Aphanius, a brief paragraph discussing it should be included (please consider credible intervals for genera and some important species-group nodes at least).

ANSWER: It was done: the following sentence was added at the end of line 343.

The bGMYC method is similar to bPTP, but it uses an ultrametric tree to delimit species. As no mutation rate has been reported for COI in genus *Aphanius*, the maximum age for *Aphanius* was set at 34 Ma based on the oldest known fossil of the Old World killifishes to produce an ultrametric tree. For this reason, and since credible intervals for some species-group nodes were broad (not shown), the bGMYC results should be cautionary interpreted.

#Finally, I recall that every manuscript should be self-contained. Please include full list of comparative material as supplementary material, so that the length of the manuscript is not increased.

ANSWER: Done. Full list of comparative materials is listed in the supplementary material:

As:

**S1 Appendix. List of comparative material examined.**

**Additional change:**

We registered and received LSID for the genus *Paraphanius* from *ZooBank* and thus the following statement was added:

**Nomenclatural acts**

The electronic edition of this article conforms to the requirements of the amended International Code of Zoological Nomenclature, and hence the new names contained herein are available under that Code from the electronic edition of this article. This published work and the nomenclatural acts it contains have been registered in ZooBank, the online registration system for the ICZN. The ZooBank LSIDs (Life Science Identifiers) can be resolved and the associated information viewed through any standard web browser by appending the LSID to the prefix “http://zoobank.org/”. The LSID for this publication is: urn:lsid:zoobank.org:pub:A8F5EA21-50CE-4FBE-969C-EA6B61FFBABE

**The mina letter sent by respected Editor**:

Dear Prof. Esmaeili,

Thank you for submitting the improved version of your  manuscript. Many suggested changes have been implemented, but some more work is needed fully meet PLOS ONE’s publication criteria. Therefore, we invite you to address all the points raised by the reviewer before re-submitting.

In particular, the parts concerning morphological analyses and fossil calibration must be changed according to the suggestions provided.  

We would appreciate receiving your revised manuscript by May 10 2020 11:59PM. When you are ready to submit your revision, log on to <https://www.editorialmanager.com/pone/> and select the 'Submissions Needing Revision' folder to locate your manuscript file.

If you would like to make changes to your financial disclosure, please include your updated statement in your cover letter.

To enhance the reproducibility of your results, we recommend that if applicable you deposit your laboratory protocols in [protocols.io](http://protocols.io/), where a protocol can be assigned its own identifier (DOI) such that it can be cited independently in the future. For instructions see: <http://journals.plos.org/plosone/s/submission-guidelines#loc-laboratory-protocols>

Please include the following items when submitting your revised manuscript:

- A rebuttal letter that responds to each point raised by the academic editor and reviewer(s). This letter should be uploaded as separate file and labeled 'Response to Reviewers'.
- A marked-up copy of your manuscript that highlights changes made to the original version. This file should be uploaded as separate file and labeled 'Revised Manuscript with Track Changes'.
- An unmarked version of your revised paper without tracked changes. This file should be uploaded as separate file and labeled 'Manuscript'.

  Please note while forming your response, if your article is accepted, you may have the opportunity to make the peer review history publicly available. The record will include editor decision letters (with reviews) and your responses to reviewer comments. If eligible, we will contact you to opt in or out.

 We look forward to receiving your revised manuscript.

Kind regards,

Roberta Cimmaruta, PhD
Academic Editor
PLOS ONE


[Note: HTML markup is below. Please do not edit.]

Reviewers' comments:

Reviewer's Responses to Questions

**Comments to the Author**

1. If the authors have adequately addressed your comments raised in a previous round of review and you feel that this manuscript is now acceptable for publication, you may indicate that here to bypass the “Comments to the Author” section, enter your conflict of interest statement in the “Confidential to Editor” section, and submit your "Accept" recommendation.

Reviewer #2: (No Response)

2. Is the manuscript technically sound, and do the data support the conclusions?

The manuscript must describe a technically sound piece of scientific research with data that supports the conclusions. Experiments must have been conducted rigorously, with appropriate controls, replication, and sample sizes. The conclusions must be drawn appropriately based on the data presented.

Reviewer #2: Yes

3. Has the statistical analysis been performed appropriately and rigorously?

Reviewer #2: Yes

4. Have the authors made all data underlying the findings in their manuscript fully available?

The [PLOS Data policy](http://www.plosone.org/static/policies.action#sharing) requires authors to make all data underlying the findings described in their manuscript fully available without restriction, with rare exception (please refer to the Data Availability Statement in the manuscript PDF file). The data should be provided as part of the manuscript or its supporting information, or deposited to a public repository. For example, in addition to summary statistics, the data points behind means, medians and variance measures should be available. If there are restrictions on publicly sharing data—e.g. participant privacy or use of data from a third party—those must be specified.

Reviewer #2: Yes

5. Is the manuscript presented in an intelligible fashion and written in standard English?

PLOS ONE does not copyedit accepted manuscripts, so the language in submitted articles must be clear, correct, and unambiguous. Any typographical or grammatical errors should be corrected at revision, so please note any specific errors here.

Reviewer #2: Yes

6. Review Comments to the Author

Please use the space provided to explain your answers to the questions above. You may also include additional comments for the author, including concerns about dual publication, research ethics, or publication ethics. (Please upload your review as an attachment if it exceeds 20,000 characters)

Reviewer #2: DNA barcoding and species delimitation of the Old World tooth-carps, family Aphaniidae Hoedeman, 1949 (Teleostei: Cyprinodontiformes), Esmaeili et al.

This is the second time I review this manuscript. I thank the authors for the modifications and changes to the ms., but I still have some recommendations that will improve the quality of the manuscript. Please consider that some of the changes were difficult to understand, either because the response was not detailed (i.e. not stating the lines modified) or simply because they were not considered.

I will add a few suggestions following the original order of comments, especially those comments that were not considered by the authors (or were not justified).

#Line 203. Change “Sampling and morphological identification” to “Sampling and external morphological identification”

Corrected: It was changed to: Sampling and external morphological identification

#If meristic counts of fin rays were not done in cleared and stained specimens, please change: “based on the external morphology, including coloration and meristic counts” to “based on the external morphology and including coloration.” (lines 207-208).

Corrected: It was changed to “based on the external morphology and including coloration”

#Then add to the end of that paragraph “Meristic counts were done externally (not using cleared and stained specimens).” (line 210).

Corrected: the following sentence was added to the end of paragraph.

“Meristic counts were done externally (not using cleared and stained specimens)”

To the knowledge of this reviewer, fin rays count should always be done in cleared and stained specimens and not externally.

#Line 275. Add “belonging to morphologically identified species based on external characters”

It was in line 220:

Corrected: belonging to morphologically identified species based on external characters”

#As I mentioned in my previous review, every manuscript should be self-contained. Please include temperature cycle and which Taq/reagents/mastermix was used for PCR (lines 223-225).

The following sentence was added:

The amplification process was performed using Master Mix in a total volume of 25 μl containing 12.5 μl of a Ready 2X PCR Master Mix (Genetbio, Cat. no. G-2000), 0.5 μl of each primer (10 pmol/μl), 5 μl of the DNA template and 6.5 μl dd water. The amplification was performed on a Bioer XP Thermal Cycler (Bioer Technology Co. Ltd., Hangzhou, China), programmed as following: an initial denaturation at 94 °C for 3 min, 35 cycles with denaturation at 94 °C for 50 s, annealing at 52 °C for 1min, and a final extension phase at 72 °C for 5 min.


#Lines 239-241. Please move the phrase “The nucleotide substitution pattern showed that the sequences have not reached substitution saturation and are therefore well applicable for phylogenetic analyses.” to results section.

Done: the phrase was moved to the results section under the heading of **Molecular species delimitation (**lines 299-300).

#In my previous review I mentioned that considering the fossil calibration used (maximum age 34 Ma) (lines 307-309), the results should be cautionary interpreted. As no mutation rate has been reported for COI in Aphanius, a brief paragraph discussing it should be included (please consider credible intervals for genera and some important species-group nodes at least).

It was done: the following sentence was added at the end of line 343.

The bGMYC method is similar to bPTP, but it uses an ultrametric tree to delimit species. As no mutation rate has been reported for COI in genus *Aphanius*, the maximum age for *Aphanius* was set at 34 Ma based on the oldest known fossil of the Old World killifishes to produce an ultrametric tree. For this reason, and since credible intervals for some species-group nodes were broad (not shown), the bGMYC results should be cautionary interpreted.

#Finally, I recall that every manuscript should be self-contained. Please include full list of comparative material as supplementary material, so that the length of the manuscript is not increased.

 Done. Full list of comparative materials is listed in the supplementary material:

As:

**S1 Appendix. List of comparative material examined.**

7. PLOS authors have the option to publish the peer review history of their article ([what does this mean?](https://journals.plos.org/plosone/s/editorial-and-peer-review-process#loc-peer-review-history)). If published, this will include your full peer review and any attached files.


If you choose “no”, your identity will remain anonymous but your review may still be made public.


**Do you want your identity to be public for this peer review?** For information about this choice, including consent withdrawal, please see our [Privacy Policy](https://www.plos.org/privacy-policy).

Reviewer #2: No


[NOTE: If reviewer comments were submitted as an attachment file, they will be attached to this email and accessible via the submission site. Please log into your account, locate the manuscript record, and check for the action link "View Attachments". If this link does not appear, there are no attachment files to be viewed.]


While revising your submission, please upload your figure files to the Preflight Analysis and Conversion Engine (PACE) digital diagnostic tool, <https://pacev2.apexcovantage.com/>. PACE helps ensure that figures meet PLOS requirements. To use PACE, you must first register as a user. Registration is free. Then, login and navigate to the UPLOAD tab, where you will find detailed instructions on how to use the tool. If you encounter any issues or have any questions when using PACE, please email us at [figures@plos.org](mailto:figures@plos.org). Please note that Supporting Information files do not need this step.

It was done.

**We got** LSID **for *Paraphanius* and thus the following statement was added:**

**Nomenclatural acts**

The electronic edition of this article conforms to the requirements of the amended International Code of Zoological Nomenclature, and hence the new names contained herein are available under that Code from the electronic edition of this article. This published work and the nomenclatural acts it contains have been registered in ZooBank, the online registration system for the ICZN. The ZooBank LSIDs (Life Science Identifiers) can be resolved and the associated information viewed through any standard web browser by appending the LSID to the prefix “http://zoobank.org/”. The LSID for this publication is: urn:lsid:zoobank.org:pub:A8F5EA21-50CE-4FBE-969C-EA6B61FFBABE
